# A Prospective, Open-Label, Randomized, Comparative, Investigator-Initiated Study to Evaluate the Safety and Effectiveness of Autologous Growth Factor Concentrate Using the Healrex Therapy Kit With Standard Wound Care in Lower-Extremity Diabetic Ulcer

**DOI:** 10.7759/cureus.75936

**Published:** 2024-12-18

**Authors:** Ketan Vagholkar, Sandesh Deolekar, Tushar Rege, Shrikant N Kurhade, Shrikant Deshpande, Sher Singh Dariya, Khokan Debnath, Salim Patel, Vijay Sharma, Anuka Sharma

**Affiliations:** 1 General Surgery, Dr. D.Y. Patil Medical College and Hospital, Navi Mumbai, Navi Mumbai, IND; 2 General Surgery, KK Care Hospital, Pune, IND; 3 General Surgery, Ashirwad Hospital, Thane, IND; 4 Internal Medicine, Sawai Man Singh Hospital, Jaipur, IND; 5 Clinical Operations, Pharmacovigilance and Regulatory, Wockhardt Ltd., Mumbai, IND; 6 Medical Affairs, Wockhardt Regenerative Private Ltd., Mumbai, IND; 7 Stem Cell Research, Wockhardt Hospitals Ltd., Mumbai, IND; 8 Business Development Hospitals, Wockhardt Hospitals Ltd., Mumbai, IND

**Keywords:** agfc, diabetic foot ulcer, growth factor, healing, standard wound care

## Abstract

Background and objectives

The persistent nature of diabetic foot ulcers (DFUs) is mainly attributable to compromised wound healing mechanisms, which are aggravated due to poor blood flow, neuropathy, and infection. Growth factors have become essential agents in the treatment of DFUs, serving as primary mediators that enhance wound healing through the stimulation of cell proliferation, migration, and angiogenesis. This prospective open-label, randomised, comparative, multi-centre, investigator-initiated study compared the safety and effectiveness of adjuvant therapy with topical application of autologous growth factor concentrate (AGFC) using the Healrex^®^ therapy kit (Wockhardt, India) versus standard of care (SoC) in DFUs.

Methods

Fifty-two adult men and women with DFU (Grades I or II as per Maggitt-Wagner classification) were randomised to the Healrex^®^ (n = 26) or SoC (n = 26) group. AGFC concentrate was prepared using the Healrex^®^ therapy kit, and application was from baseline to day 70 (visit 2 to visit 16). Wound assessment and size estimation post debridement were done from screening, i.e., day -03 to -01 day to 70 days (visit 1 to visit 16). The primary outcome was complete response defined as the proportion of patients having healthy granulation tissue covering ≥75% of the ulcer surface at the test of cure (ToC) or visit 17, whereas secondary outcomes were the percentage of patients with complete wound closure at ToC/visit 17, mean percentage reduction of ulcer size at ToC/visit 17 and time to appearance of healthy granulation tissues. Safety was measured in the form of treatment-emergent adverse events (TEAEs) reported, deviations in the desirable range of parameters determined using laboratory tests (haematology, serum biochemistry, urine examination), and electrocardiogram (ECG) done from baseline to ToC. Two patients were lost to follow-up, and one from the Healrex^®^ arm was withdrawn from the study, resulting in a final efficacy analysis on 49 participants (24 in the Healrex^®^ group and 25 in the SoC group) in the per-protocol (PP) dataset. Safety analysis was conducted on the intention-to-treat (ITT) dataset, which included all 52 participants.

Results

Complete response was observed in all 24 patients (n = 24 (100.0%)) with Healrex^®^ compared to only n = 21 (84.0%) with SoC (p = 0.042). Complete wound closure was observed in 11 patients (n = 24 (45.8%)) treated with Healrex^®^ compared to 13 patients (n = 13 (52.0%)) with SoC. The time required for wound closure was similar (p = 0.669) in the two groups. A greater reduction in the ulcer area was observed with Healrex^®^ as against SoC (p < 0.0001). The time for the emergence of healthy granulation tissue was comparable between the two groups (p = 0.342). Ten patients with Healrex^®^ reported mild adverse events (headache, fever, and cold) and none with SoC.

Conclusion

AGFC application using the Healrex^® ^therapy kit as an adjuvant to standard wound care provides better outcomes compared to SoC alone in the management of DFUs.

## Introduction

A diabetic foot ulcer (DFU) is a full-thickness wound involving the dermis in either the ankle or foot with a 25% lifetime risk [1]. DFUs are among the most challenging complications for patients who have diabetes mellitus, which is not well controlled. Around 15-25% of patients with diabetes mellitus will develop DFUs during their lifetime. DFU is associated with significant morbidity, and the mortality ranges between 4% and 10%. Zhang et al. reported a 6.3% global prevalence of diabetic foot in a meta-analysis [2]. It is also one of the common causes of osteomyelitis of the foot and amputation of the lower extremities [3]. Loss of a limb leads to enormous morbidity, and many patients are not able to afford a prosthesis. Most remain disabled for life and lead a poor quality of life. DFUs and related sepsis were found to be the second most common form of infection-related mortality (8.3%) in hospitalised patients [4]. DFUs contribute to approximately 80% of all non-traumatic amputations in India annually. Patients with a history of DFU have a 40% higher 10-year death rate than those without. The average time required for healing of DFUs is 28 weeks (range 12 to 62 weeks) [5].

Despite growth factors being recognised as essential signalling molecules in healing, their utilisation in surgery is still constrained. In the last few years, the most effective preparations have been autogenously sourced, including platelet-rich plasma (PRP) and platelet-rich fibrin (PRF). Despite advances in wound care, the healing of DFUs remains problematic, often leading to prolonged treatment durations, high rates of infection, and an increased risk of amputation. Effective management of DFUs is essential to improving patient outcomes and reducing the overall burden of diabetic complications. The treatment of DFU requires an immediate decision and systematic approach that comprises maintaining arterial blood flow, treating the infection appropriately, and removing the pressure from the wound [6]. In the past decade, many novel and basic science-based approaches and developments for adjuvant therapies including wound dressing, hyperbaric oxygen therapy, or growth factor formulations for efficient local delivery [7-9]. The standard care for DFUs typically involves debridement, infection control, and dressings that maintain a moist wound environment. While these methods can promote healing, there is a continuous need for innovative therapies that can accelerate wound healing and enhance the quality of life for patients with DFUs [10]. Autologous growth factor concentrates (AGFCs) derived from the patient's blood have emerged as a promising treatment option. Growth factors play a crucial role in the wound-healing process by promoting cellular proliferation, angiogenesis, and tissue regeneration [11]. The Healrex® therapy kit enables the preparation and application of AGFCs, potentially enhancing the healing of chronic wounds such as DFUs.

This research was undertaken due to a notable scarcity of studies examining the safety and effectiveness of AGFCs in DFUs. The lack of existing literature highlights the need for a thorough investigation to provide valuable insights and inform clinical practice. This study compared the effectiveness and safety of topical application of AGFCs using the Healrex® therapy kit along with standard wound care versus standard wound care (SoC) in patients with DFUs. This study provides critical data on the potential benefits of AGFCs in promoting the appearance of healthy granulation tissue, complete wound closure, time to appearance of healthy granulation tissue, and overall reduction in ulcer size.

## Materials and methods

Methodology

Study Design and Setting

This prospective, open-label, randomised, multi-centre, investigator-initiated study (IIS) was conducted at six clinics across India. The study conducted was as per the Good Clinical Practice guidelines (ICH-GCP E6), New Drugs and Clinical Trials Rules (2019), and Declaration of Helsinki (Taipei 2016). The study was pre-registered with the Clinical Trials Registry of India (CTRI/2023/03/050481 dt: 09/03/2023). The study documents were reviewed and approved by the Institutional Ethical Committees of all centres: Royal Pune Independent Ethics Committee (RPIEC C061023, dt:11 Oct 2023); Dr. D. Y. Patil Medical College, Hospital and Research Centre, Navi Mumbai (DU)/IEC/ 01-A/2023 dt.16 Jan 2023); Tagore Hospital Ethics Committee (THEC2024/01/01); and Ashirvad Ethics Committee Ulhasnagar, Maharashtra (AEC dt: 14 Oct 2023). Informed written consent was obtained from all participants in their preferred language prior to enrolment. Each participant was explained in detail about the study objective and the expected outcome before obtaining consent.

Study Participants

Adults (18 years or older) of any sex with type 1 or type 2 diabetes mellitus and controlled blood glucose levels, who had a single chronic ulcer that has persisted for at least one month despite appropriate wound care, were eligible to participate in the study. Other inclusion criteria were as follows: The ulcer is classified as Grade I or II according to the Maggitt-Wagner classification system, where Grade I involves superficial ulceration of the skin or subcutaneous tissue, and Grade II involves a deep ulcer extending to ligament, tendon, joint capsule, bone, or deep fascia without abscess or osteomyelitis. The ulcer size ranges from 1 cm² to 25 cm² after debridement, with a well-controlled infection that does not require a skin graft. Adequate arterial blood supply confirmed by colour Doppler ultrasonography or a palpable pulse in the dorsalis pedis or posterior tibial artery. Female participants surgically sterile, post-menopausal, or agreed to use adequate contraception and have a negative pregnancy test at screening and must not be nursing were included. In addition, patients who were able and willing to provide informed consent and comply with protocol visits and procedures were included. Patients who were free from interdigital ulcers or wounds resulting from an amputation site, ulcers induced by electrical, chemical, or radiation injuries, pressure ulcers, vascular ulcers, or Charcot deformities were excluded. Other exclusions were active ulcer infections evaluated through clinical examination and, if required, radiography, as well as active osteomyelitis in the ulcer vicinity, indicated by necrosis, purulence, or sinus tracts that were unmanageable through debridement and standard wound care. Patients exhibiting poorly managed diabetes (HbA1c% ≥10%), renal insufficiency (serum creatinine >2.0 mg/dL), inadequate nutritional condition (albumin <3.0 g/dL or total protein <6.5 g/dL), thrombocytopenia (platelet count <100,000/dL), haemoglobin levels <10 gm/dL, and random blood sugar levels >200 mg/dL were excluded. Individuals who were positive for hepatitis B or C, HIV, or reactive VDRL/RPR, as well as those with established connective tissue or malignant disorders, or a history of serious cerebrovascular incidents, were also excluded. Patients undergoing concurrent treatment with corticosteroids, immunosuppressive drugs, radiation therapy, or anticancer chemotherapy or those who have utilised an investigational drug/device or growth factor within 30 days prior to screening were ineligible. Prior treatment utilising advanced therapies for lower-extremity diabetic ulcers, such as negative-pressure wound therapy (NPWT) devices, hyperbaric oxygen, or growth factors within seven days after screening were also ineligible. Furthermore, individuals with blood dyscrasias, people receiving anticoagulant or antiplatelet therapy, individuals with active bleeding wounds or hemoglobinopathies, pregnant or lactating ladies, and fertile women not utilising contraception were precluded. Patients anticipated to be non-compliant with the protocol or considered unfit by the investigator for any other reason were ineligible.

Randomisation and Blinding

Enrolled patients were assigned to either the Healrex® arm or SoC arm in a 1:1 ratio. Randomisation was done using a computer-based pre-determined randomisation chart. Being an open study, randomisation was concealed till the enrolled participants were assigned a study participant number (ID). After the study number was assigned to a participant, the sealed envelopes for the participant were opened to reveal the treatment to be received by the participant.

Interventions

Healrex® arm: AGFC derived from the Healrex® (Wockhardt, India) therapy kit along with standard care including debridement or removal of necrotic tissue, wound cleansing, and dressings that promote a moist wound environment.

SoC (reference) arm: Topical application of normal saline with SoC including debridement or removal of necrotic tissue, wound cleansing, and dressings that promote a moist wound environment, e.g., moist wound dressing/saline gel/hydrogel.

Patients continued their regular medication for diabetes or any other conditions. The decision to initiate or continue antibiotic treatment was at the investigator's discretion. All patients received adequate glycaemic management.

AGFC Preparation from the Healrex® (Wockhardt, India) Therapy Kit

At day -03 to -01, screening procedures were conducted, including obtaining written informed consent, assessing demographic information, past medical and surgical history, physical examination, vital sign monitoring, wound assessment, and laboratory investigations. Subsequent visits from day 5 (±2 days) to day 70 (±2 days), i.e., end of treatment (EoT) visit involved physical examination, vital sign monitoring, wound care procedures including debridement and infection control, assessment of wound and granulation tissue formation, topical application of the assigned treatment, monitoring of adverse events, and concomitant medication.

Approximately 25 ml of blood was collected on day 0, day 25 ± 2 days, and day 50 ± 2 days for the preparation of AGFCs. After blood collection, the GFC kit tubes were allowed to stand for 30 minutes. They were then centrifuged at 3400 rpm for 10 minutes, ensuring they were counterbalanced. Post-centrifugation, the GFC was separated above the gel, with other cellular components below. The GFC from one tube was used for dressing on the same day by inverting the tube to bring the GFC to the cap and extracting it with a 5 ml syringe. The GFC in the syringe was used immediately or within four hours if refrigerated at 2-8°C. Each tube yielded approximately 1.5 to 2 ml of GFC. The GFC from the remaining four tubes was collected similarly and transferred into separate red top tubes provided in the kit. These red top tubes were stored in the freezer. They were thawed at room temperature for 15-20 minutes before application during wound redressing. The thawed GFC was applied to the clean wound surface. Dressing was recommended on every fifth day (±2 days) with the GFC till the wound healing process was complete. One tube/vacutainer GFC was used for every application on a maximum of 5 cm^2^ wound.

Study outcomes

Primary Outcome

The primary outcome of the study was the percentage of subjects having a ‘complete response’ on day 75, i.e., test of cure (ToC). Complete response was defined as the appearance of healthy granulation tissue covering ≥75% of the ulcer area. Healthy granulation tissue was defined as tissue with serous exudate, fine granules, bright red colouration, and bleeding upon touch. Baseline ulcer size was measured from margin to margin of the ulcer using head-to-toe orientation. Ulcer size (surface area) = longest length × greatest width perpendicular to the length (using head-to-toe orientation). The percentage of subjects with healthy granulation tissue was calculated, and a two-sided exact 95% confidence interval was computed to assess the precision of the estimate.

Secondary Outcomes

The secondary outcomes included the percentage of patients with complete wound closure at ToC/visit 17, mean per cent reduction in the ulcer size (surface area) at ToC visit and time to appearance of healthy granulation tissue covering ≥75% of the ulcer surface. The proportion of patients achieving complete wound closure of DFUs was evaluated at ToC. Complete wound closure was defined as 100% epithelisation, normal colouration with no marginal recurrence, complete absence of exudate, and no clinical signs of infection.

Safety Outcomes

Any adverse event emerging during the study, including worsening relative to the pre-treatment state, was monitored and recorded. A 12-lead electrocardiogram (ECG) and laboratory assessments were done from baseline to ToC on blood and urine to assess the safety of AGFCs in lower-extremity diabetic ulcers. Laboratory tests also included blood chemistry (alanine aminotransferase, aspartate aminotransferase, total protein, serum electrolytes (Na+, K+, Cl-), partial thromboplastin time, international normalised ratio, and serum creatinine).

Sample Size

It was planned to enrol 50 participants (satisfying 90% power with 10% dropout) after considering the inclusion and exclusion criteria. A total of 52 patients were randomised, and 49 patients were considered for final efficacy analysis.

Statistical methods

The primary efficacy endpoint of the study is the percentage of subjects with the appearance of healthy granulation tissue. The percentage was computed with a two-sided, exactly 95% CI. All other efficacy and safety endpoints were analysed using e-counts and percentages (frequency).

In the analysis described, two primary statistical approaches were utilised to assess the effects of treatment over time while adjusting for baseline characteristics. First, a generalised linear model (GLM) was employed to evaluate the impact of treatment while accounting for time as a repeated measures factor and baseline vital parameters as covariates. This approach allowed for the estimation of adjusted scores on different scales, effectively isolating the treatment effect by controlling for the influence of baseline vital parameters. This technique helps ensure that any observed differences in outcomes can be attributed to the treatment itself rather than variations in baseline characteristics. Second, a repeated measures analysis of variance (ANOVA) was performed to analyse changes over time in several dependent variables, including pain (measured by the visual analogue scale (VAS) score), tenderness grade, wound assessment, and granulation tissue. In this analysis, time (visit day) was treated as a repeated measures factor to account for multiple observations from the same subjects, while the treatment group served as the independent variable to evaluate its effect. This method assesses whether there are significant differences in these outcomes both over time and between different treatment groups, providing a comprehensive understanding of treatment effects and temporal changes in the measured variables.

## Results

**Figure 1:**
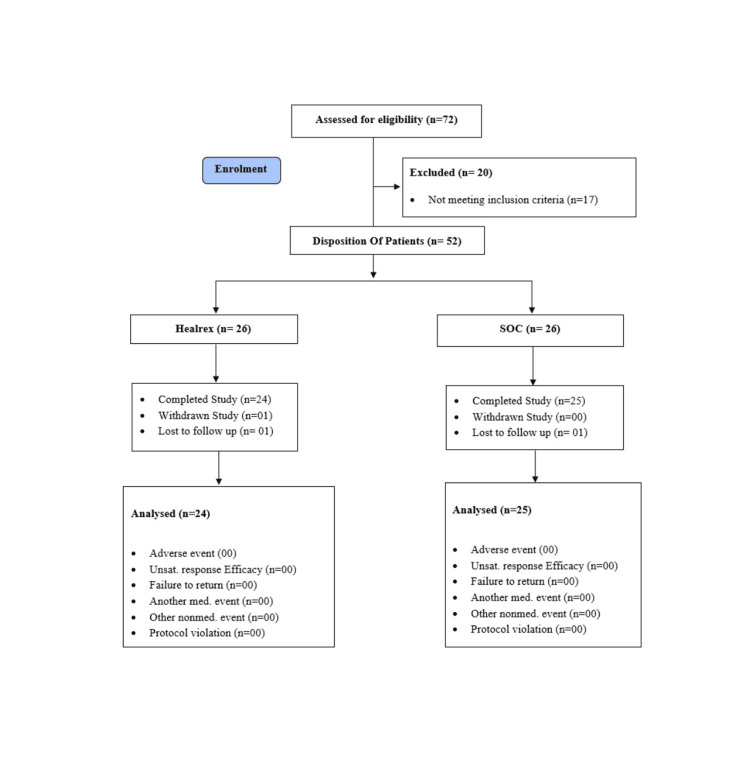
Disposition of the patients n = number of patients

Out of 72 patients screened for eligibility, 20 did not meet the criteria, leaving 52 patients who were randomised into two groups: 26 in the Healrex® arm and 26 in the SoC arm (see disposition of patients, Figure 1). Two patients were lost to follow-up, and one from the Healrex® arm was withdrawn from the study, resulting in a final efficacy analysis on 49 participants (24 subjects in the Healrex® group and 25 subjects in the SoC group) in the per-protocol (PP) dataset. Safety analysis was conducted on the intention-to-treat (ITT) dataset, which included all 52 participants. Table 1 presents the demographic data of subjects in the ITT dataset (n = 52).

**Table 1:** Demography and baseline profile of the patients in the ITT dataset (n = 52) The data has been represented as No., % and Mean ± SD No.: number of patients; yrs.: years; BMI: body mass index; SD: standard deviation; ITT: intention-to-treat

Parameter	Healrex^®^ (n = 26)	SOC (n = 26)	Total (n = 52)
Demography	Mean (SD)	Mean (SD)	Mean (SD)
Age (yrs.)	56.92 (9.01)	58.96 (12.35)	57.94 (10.75)
BMI (kg/sq.m2)	25.31 (3.65)	25.97 (4.18)	25.64 (3.90)
Sex	No. (%)	No. (%)	No. (%)
Male	15 (57.7%)	22 (84.6%)	37 (71.20%)
Female	11 (42.3%)	4 (15.4%)	15 (28.80%)
Hospitalization status	No. (%)	No. (%)	No. (%)
Hospitalized	4 (15.4%)	0 (0.00%)	4 (7.70%)
Not hospitalized	22 (84.6%)	26 (100.00%)	48 (92.30%)

Primary outcomes

Table 2 depicts the complete response rates, defined as granulation tissue covering at least 75% of the wound surface area. Twenty-four (n = 24, 100%) subjects in the Healrex® arm achieved the complete response rate, while 21 (n = 21, 81%) of the subjects achieved a complete response rate in the SOC arm at ToC. The relative risk (RR) for a complete response was 1.190 (95% CI: 1.003 to 1.412; p = 0.042), favouring Healrex®.

**Table 2:** Complete response and wound closure at ToC in the PP dataset (n = 49) The data has been represented as No., %, RR and 95% C.I. for RR. No.: number of patients; C.I.: confidence interval; SD: standard deviation; SoC: standard of care; RR: relative risk; p: p-value; ToC: test of cure; PP: per-protocol A p-value of <0.05 suggests significant differences between the groups.

Outcome parameter	Healrex^®^ (n = 24)		SoC (n = 25)		Risk ratio		
	No.	%	No.	%	RR	95% C.I. for RR	P
Response type							p=0.042*
Complete response	24	100.0%	21	84.0%	1.190	1.003 to 1.412	
No response	0	0.0%	4	16.0%			
Wound closure							p=0.667
Complete wound closure	11	45.8%	13	52.0%	0.881	0.496 to 1.567	
No wound closure	13	54.2%	12	48.0%			

Secondary outcomes

Table 2 presents the percentage of patients with complete wound closure at ToC. In the Healrex® arm, 11 patients (n = 11, 45.8%) achieved complete wound closure, compared to 13 patients (n = 11, 52%) in the SOC arm. With a p-value of 0.669, the difference in wound closure rates between the two groups was not statistically significant.

Table 3 presents the mean (SD) percentage reduction in ulcer size (surface area) at ToC. The mean (SD) percentage reduction in ulcer size was 91.57 (5.75) cm^2^ for the Healrex® arm and 82.91 (9.77) cm^2^ for the SoC arm. The reduction in ulcer size was significantly greater (p < 0.0001) with Healrex® compared to SoC. Table 3 also presents the time (in days) to the appearance of healthy granulation tissue covering at least 75% of the ulcer surface area on day 75. The mean (SD) time for this to occur was 75.00 (0.00) days for the Healrex® arm and 73.85 (5.88) days for the SoC arm. The time required for the appearance of healthy granulation tissue was similar between Healrex® and SoC (P = 0.960).

**Table 3:** Ulcer size and time (in days) to the appearance of healthy granulation tissue at ToC in the PP dataset (n = 49) The data has been represented as N, %, Mean ± SD, difference in mean, 95% C.I. of difference in mean, t-value, and p-value. N: number of patients; Mean diff. : difference in mean; C.I. of diff.; confidence interval of difference in mean; SD: standard deviation; SoC: standard of care; t: t-value; p: p-value; ToC: test of cure; PP: per-protocol

Outcome parameter	N	Mean	SD	Mean diff.	95% C.I. of diff.		t-test	
					Lower	Upper	t	p
Percentage reduction of ulcer size (surface area in sq. cm)								
Healrex^®^ (sq. cms)	24	91.57	5.75	8.66	4.03	13.30	3.762	<0.0001*
SoC (sq. cms)	25	82.91	9.77					
Time (in days) to appearance of granulation tissue								
Healrex^®^ (days)	24	75.00	0.00	1.15	-1.26	3.57	0.960	0.342
SoC (days)	25	73.85	5.88					

At baseline, mean pain scores (On 0-10 VAS) between the subjects in the Healrex® and SOC groups were similar (8.08 vs. 8.52; p = 0.409). At ToC, the subjects in the Healrex® group showed significantly lower mean pain scores (1.45 vs. 2.50; p = 0.003) compared to subjects in the SoC group. Overall, Healrex® was more effective in reducing pain.

Safety outcomes

Out of 26 subjects in the Healrex® arm, adverse events were reported in 10 patients (n = 10, 38.5%). The adverse events consisted of headache in five patients (n = 5, 19.2%), fever in four patients (n = 4, 15.4%), and cold in one patient (n = 1, 3.8%). All the events were mild to moderate in severity and were completely resolved. There were no adverse events that were serious or that led to discontinuation of the study. Out of 26 subjects in the SOC arm, no adverse events were reported.

## Discussion

The management of DFUs is usually complex and challenging to clinicians in clinical practice. Costs of DFUs have been increased to the treatment cost of many common cancers. Surgical debridement followed by frequent dressing changes along with tight infection and glycaemic control are the standard management procedures. Complication and amputation rates remain high despite this comprehensive approach. Therefore, foot ulcers should be treated immediately by a multidisciplinary expert team for optimal outcomes.

Growth factors play a crucial role as the primary immediate mediators in wound healing [12]. Several meta‐analyses have suggested that the administration of a number of different growth factors significantly improves DFU healing compared with control alone [9,13,14]. Growth factors are crucial in facilitating wound healing and have consequently been evaluated as therapies for DFUs. Thus, along with wound care, growth factors serve as the principal immediate mediators of wound healing [12].

It has been demonstrated that the use of recombinant growth factors can replicate cell migration, proliferation, and differentiation in vivo, enabling external control of the healing process. Thus, the application of growth factors in wound healing has been well investigated [15]. Berlanga-Acosta et al. (2024) [16] examined the efficacy of epidermal growth factor (EGF) in mitigating wound chronicity and facilitating enduring healing, especially in diabetic ulcers. These studies corroborate our findings, illustrating the efficacy of growth factors, such as those in the Healrex® therapy lit, in enhancing wound healing outcomes.

In this prospective, open-label, randomised, comparative study, we compared the effects of daily topical application of the AGFC Healrex® kit against the standard wound care management in DFUs. We observed significant improvement in the efficacy parameters. Complete response was observed with Healrex® (100%) in all patients as compared to only 84.0% of patients with standard wound care (p = 0.06). In the Healrex® arm, 45.8% of patients achieved complete wound closure, compared to 52.0% of patients in the SoC arm (p = 0.669). The difference in wound closure rates between the two groups was not statistically significant. The mean percentage reduction in ulcer size was significantly greater (p < 0.0001) with Healrex® compared to SoC, whereas the time required for the appearance of healthy granulation tissue was similar between Healrex® and SoC.

There is evidence suggesting that healing in DFUs is dependent on growth factors, and their topical application can accelerate wound healing when combined with conventional wound care [17]. The review by Akingboye AA et al. (2010) highlighted the benefits of autologous-derived growth factors in DFU therapy [17]. The authors also stressed the importance of the procedures and techniques used to derive growth factors from patients’ plasma and the need for a standardised protocol. Wound healing techniques involve tissue engineering and the use of biological science to develop GFCs. The Healrex® therapy kit, developed by Wockhardt (India), is specifically designed to promote tissue growth at the ulcer base and enhance healing rates. This therapy introduces cells that release specific growth factors, thereby improving wound healing. A total of 10 patients reported adverse events in the Healrex® group during the study, whereas no adverse events were reported by the patients in the SOC group. Kakagia et al. (2007) found no complications or side effects in any patients with lower-extremity diabetic ulcers managed with autologous growth factors during the follow-up period of eight weeks [18].

Overall, this study demonstrates that the topical application of Healrex® therapy for lower-extremity diabetic ulcers led to superior clinical outcomes compared to the topical application of normal saline with SoC with lower complication rates.

Conducting this study sets the stage for further investigations into AGFC and its applications across various medical conditions. Establishing preliminary data on safety and efficacy can encourage larger-scale trials and foster interest among researchers and clinicians alike. This foundational work is essential for developing standardised treatment protocols and exploring the broader implications of AGFC in regenerative therapies.

This study fills a significant gap in the literature regarding the efficacy and safety of AGFC compared to the SoC. Given that AGFC is a relatively new approach, this study provides crucial insights into its potential benefits for the formation of healthy granulation tissue covering, reduction of ulcer size, and complete wound closure. The findings may lead to a better understanding of how AGFC can enhance treatment outcomes in DFU, thus contributing to the advancement of regenerative medicine.

This study had a sample size of 52 participants, which may limit the generalisability of the findings. This limitation can be addressed by conducting further studies with larger sample sizes and extended follow-up periods, which are recommended to confirm these findings and improve their generalisability.

## Conclusions

The study demonstrates that the Healrex® AGFC therapy kit, when used in conjunction with standard wound care, is effective in promoting wound healing in lower-extremity diabetic ulcers, with a higher complete response rate and greater reduction in ulcer size compared to standard care alone. The safety profile of Healrex® was also favourable, with no serious adverse events reported. These findings support the potential of Healrex® as a beneficial treatment option for patients with DFUs. Further studies with larger sample sizes and longer follow-up periods are recommended to validate these findings.
